# Effect of vitamin B12 on the symptom severity and psychological profile of fibromyalgia patients; a prospective pre-post study

**DOI:** 10.1186/s41927-022-00282-y

**Published:** 2022-09-01

**Authors:** Faeze Gharibpoor, Banafsheh Ghavidel-Parsa, Nazila Sattari, Ali Bidari, Fatemeh Nejatifar, Ali Montazeri

**Affiliations:** 1 Student Research Committee, Faculty of Medicine, Guilan University of Medical Sciences, Rasht, Iran; 2 Department of Rheumatology, School of Medicine, Rheumatology Research Center, Razi Hospital, Guilan University of Medical Science, Sardar Jangal St, Rasht, 41448-95655 Guilan Iran; 3 Department of Rheumatology, Iran University of Medical Sciences, Tehran, Iran; 4 Department of Hematology and Medical Oncology, Guilan University of Medical Sciences, Rasht, Iran; 5 Population Health Research Group, Health Metrics Research Center, Iranian Institute for Health Sciences Research, ACECR, Tehran, Iran

**Keywords:** Vitamin B12, Cyanocobalamin, Methylcobalamin, Fibromyalgia, Chronic pain

## Abstract

**Background:**

Fibromyalgia (FM) as a prototypical nociplastic pain condition displays a difficult therapeutic situation in many cases. Given the promising data on the effect of vitamin B12 in improving pain and cognitive functions in various nociplastic pain conditions, we aimed to determine the efficacy of 1000 mcg daily dose of oral vitamin B12 on the symptom severity and psychological profile of FM patients.

**Methods:**

This open-label, pre-post study was performed on FM patients whose diagnoses were confirmed by a rheumatologist based on the 2016 American College of Rheumatology (ACR). Patients were instructed to take a daily dose of 1000mcg vitamin B12 for fifty days. Outcome measures including the Revised Fibromyalgia Impact Questionnaire (FIQR), Hospital Anxiety and Depression Scale (HADS), 12-item Short-Form health survey (SF-12), and pain Visual Analog Scale (pain-VAS) were fulfilled by patients before and after the treatment.

**Results:**

Of 30 eligible patients, 28 patients completed the study protocol. Patients were female with a mean age of 47.50 ± 8.47 years. FIQR scores in all domains improved significantly after treatment (total FIQR: 49.8 ± 21.86 vs 40.00 ± 18.36, *p* value < 0.01; function: 13.17 ± 7.33 vs 10.30 ± 5.84, *p* value: 0.01; overall: 10.32 ± 6.22 vs 8.25 ± 6.22, *p* value: 0.03; symptoms: 26.30 ± 10.39 vs 21.44 ± 8.58, *p* value < 0.01). Vitamin B12 also improved anxiety scores from 9.33 ± 4.30 to 7.70 ± 3.60, *p* value: 0.01. Depression, pain-VAS, and SF-12 didn’t improve following the treatment. The Generalized estimating equations (GEE) analysis showed the improvement in total FIQR score is not cofounded by the improvement of anxiety and patients’ baseline characteristics.

**Conclusions:**

This study showed a short course of sublingual vitamin B12, 1000 mcg daily, significantly improves the severity of FM and anxiety score. We postulate that vitamin B12 has a strong potential to consider, at least, as adjunctive therapy of FM.

**Trial registration:**

The study protocol was approved by the ethics committee of Guilan University of Medical Sciences (IR.GUMS.REC.1400.197) in accordance with the World Medical Association’s code of ethics (Declaration of Helsinki, revised in Brazil 2013), and registered at an ICMJE and WHO recognized registry of clinical trials (www.irct.ir) on 28/08/2021 (registration number: IRCT20200920048782N1).

## Introduction

Fibromyalgia (FM) is a common syndrome characterized by generalized body pain accompanied by cognitive and mood disturbances, fatigue, sleep problems, and other somatosensory symptoms [1]. FM is a challenging disease regarding its diagnosis, pathophysiology, and treatment. Although the exact pathophysiology of the disease has remained unknown, central nervous system (CNS) sensitization has been proposed as the prominent or exclusive pathway in FM pathogenesis [2]. Central sensitization, which refers to the amplification of pain by CNS mechanisms, presents clinically as pain hypersensitivity and allodynia (feeling pain in response to normally nonpainful stimulus) [3]. This hypothesis has been strongly supported by neurochemical and functional brain imaging studies which have been rapidly accumulating in the FM research field. The levels of pain inhibitory neurotransmitters such as noradrenergic and serotoninergic neurotransmitters in the biological fluid of patients with FM are lower than healthy individuals [4]. Also, proton spectroscopy which can probe specific neurotransmitters involved in neuroplasticity has displayed increased glutamatergic activity and decreased GABAergic activity in key pain related regions such as the insula [5]. The imbalance of neurotransmitters in favor of the excitatory ones such as glutamate leads to pain central sensitization [4, 5].

The cobalamin or vitamin B12 as a cofactor in the activation of numerous metabolic processes and metabolism of neurotransmitters, lipids, and proteins seems to have a fundamental role in maintaining neuronal plasticity [6]. Recent data suggest vitamin B12 as a potential micronutrient of pain killers. The preclinical data have demonstrated the neuronal protection role of vitamin B12 by promoting regeneration of injured nerves and antagonizing glutamate-induced neurotoxicity [7, 8]. The other effects of vitamin B12 on neuroplasticity and pain neurobiology may include the moderation of neurotransmitter imbalance toward decreased glutamatergic and increased GABAergic activity, interactions with prostaglandin synthesis such as cyclooxygenase (COX) enzymes, and some other complex interaction [9, 10]. Furthermore, accumulating clinical data supports the pain-relieving role of vitamin B12 in various nociceptor or neuropathic pain conditions such as low back pain, diabetic neuropathy, chemotherapy-induced pain in breast cancer patients, and postherpetic neuralgia [7, 11, 12]. However, despite numerous positive data on pain reliving effect of vitamin B12, there are scarce interventional studies about the effect of vitamin B12 on nociplastic pain.

FM as a prototypical nociplastic pain condition displays a difficult therapeutic situation in many cases. Few existing data on the serum and cerebrospinal fluid (CSF) levels of vitamin B12 are contradictory but most of them have implied no alteration of vitamin B12 levels in FM patients rather than healthy subjects [13–16]. Although Regland et al. showed a promising response rate of the Fibro Fatigue scale and Patient’s Global Impression to vitamin B12 in FM patients, there is no other study on this issue [17]. Given the high rates of psychological comorbidities in FM and with the knowledge of a higher risk of developing psychological illness in vitamin B12 deficiency, it would be reasonable to evaluate the effect of vitamin B12 on the psychological impact of FM. Although some studies have shown improved cognitive function and depression after vitamin B12 supplementation, there is no concert evidence showing positive effects of vitamin B12 on psychological illness [18, 19].

Considering the marked safety and potential positive effect of vitamin B12 on neurobiology and clinical improvement of FM, we sought to evaluate whether daily supplementation of vitamin B12 can reduce the severity of FM and improve psychological symptoms of FM patients.

## Methods

### Design and setting

This was an open-label pre-post study on a single-arm intervention group of 30 female FM patients admitted to the FM clinic of Razi academic hospital affiliated to Guilan University of Medical Sciences (GUMS) in august 2021.

The study protocol was approved by the ethics committee of Guilan University of Medical Sciences (IR.GUMS.REC.1400.197) in accordance with the World Medical Association’s code of ethics (Declaration of Helsinki, revised in Brazil 2013), and registered at an ICMJE and WHO recognized registry of clinical trials (www.irct.ir) on 28/08/2021 (registration number: IRCT20200920048782N1). All participants were informed about the study design and then written informed consent was obtained from all volunteers. They were informed that their level of care wouldn’t be affected if they discontinued the study.

### Participants

The diagnosis of FM was made by one rheumatologist (B.Gh) according to the 2016 American College of Rheumatology (2016 ACR) criteria [20]. Patients were excluded if they were under 18 years old, pregnant or breastfeeding, suffering from any comorbidity with nociceptive or neuropathic chronic pain or inflammation (e.g., major recent trauma, malignancy, other non-inflammatory or inflammatory rheumatic diseases, celiac disease, inflammatory bowel disease, and any kind of neuropathic pain). Regarding the previous studies implying the normal level of vitamin B12 in FM patients [13], we forbore the checking of vitamin B12 level in this study; however, in order to avoid inclusion of patients with vitamin B12 deficiency, we excluded patients with a history of gastrectomy or bypass surgery and a vegetarian diet.

### Intervention

At the initial visit, patients were evaluated for demographic data (including age, marital status, education level, occupation, time from their first symptoms, time to a confirmed diagnosis, and prior medications) and outcome measures at baseline and day 50 ± 2. Outcome measures included revised fibromyalgia impact questionnaire (FIQR), Hospital anxiety and depression scale (HADS), 12-item short-form health survey (SF-12), and pain visual analog scale (pain-VAS). Two medical students (F.GP and N.S) helped patients to fulfill the questionnaires. Patients were instructed to take sublingual pearl of vitamin B12, 1000mcg once daily for 50 days, 30 min before or 2 h after taking food. Since there was no consensus regarding vitamin B12 dosage, we chose the mentioned dosage as it was commercially available in our country [21]. Patients were instructed to keep a 6 h time gap between the proton pump inhibitor (PPI) if consumed, and the vitamin. Patients taking prior medication to relieve FM symptoms were encouraged to continue their regimen. Patients who were already taking any supplement containing vitamin B12 were asked to stop taking them for a minimum of 2 weeks before the study. An intake calendar was given to patients to track their compliance. The patients were also informed to report any unpredicted symptoms at any time during the study.

### Outcome measures

The FIQR is a 21-items questionnaire with an 11-point numeric rating scale for each question which assesses clinical symptom severity and disease impact in patients with FM [22]. The total score of the FIQR ranges between 0 and100 with a higher score indicating worse disease impact. FIQR has three domains evaluating patients’ function, overall impact, and symptoms severity. The SF-12 questionnaire evaluates the health status including the mental and physical health domains with 8 scales. Scores range from “0 to 100” where “0” indicates the worst condition and “100” indicates the best possible condition [23].

HADS is a 14 items questionnaire with two subclasses: 7 questions for anxiety and 7 questions for depression. Each question scores between 0 and 3, with a maximum score of 21 for each subclass. Higher scores mean a higher level of anxiety and depression [24].

### Sample size calculation

The sample size was calculated using G*Power 3.1 [25]. Since there was no prior pre-post study on the administration of vitamin B12 on FM patients, we obtained pain scores from a previous study by Campbell et al., who measured the worst pain score, before (7.73 ± 1.74) and after (5.61 ± 2.56) application of oral vitamin B12 in chemotherapy-related pain in breast cancer patients [12]. Type 1 error (two-tailed) was considered 5% with 80% power and correlation between two groups (before and after treatment) of 0.5. The calculated effect size was 0.9 which was reduced to 0.8. The minimum sample size with 30% dropout was 21 patients.

### Statistical analysis

Descriptive statistics were used to calculate the mean and standard deviation for continuous variables and frequency for categorical variables. Shapiro–Wilk test was used to test the normality of data and Levene’s test was used for the equality of variances. Paired t-test was used to compare the outcomes of each group (except for the mental component of SF12) since the difference between paired samples was normally distributed. Wilcoxon test was used for comparing the mental component of SF12.

Generalized estimating equations (GEE) was used to develop models to test whether a change in FIQR score after treatment (shown by variable time) is associated with other variables. The first model assesses whether the effect of vitamin B12 on FIQR score (shown by the variable time) remains significant after adjusting for the change in anxiety. The second model investigates whether a change in FIQR score remains significant when adjusted for age, time from diagnosis marital status, and baseline level of depression and anxiety. We used Quasi Likelihood under Independence Model Criterion (QIC) to choose the right working correlation matrix structure. In the analysis of all models, “Unstructured” was used as the correlation structure.

All statistical tests were two-tailed, and p values < 0.05 were considered significant. Statistical calculations were performed using the IBM SPSS Statistics for Windows, version 26 (IBM Corp., Armonk, N.Y., USA).

## Result

### Population characteristic

Of 30 patients receiving vitamin B12, 28 patients completed their treatment course. One patient was lost for follow up and one patient wasn’t compliant with following the study protocol. No adverse effect was reported. Most patients were married, unemployed and more than half of patients didn’t have a university education. A detailed description of participants is available in Table 1.

**Table 1:** Baseline patient characteristics

Variable	Value
Age, years (Mean (SD))	47.50 (8.47)
*Education, years, percent (number)*	
0–12 12–16 > 16	57.1% (16) 35.7% (10) 7.1% (2)
*Marital status, percent (number)*	
Single Married	7.1% (2) 92.9% (26)
*Employment status, percent (number)*	
Unemployed Employed	75% (21) 25% (7)
Time from diagnosis, years (median (SD))	2.0 (2.98)
Onset of symptoms, years (median (SD)	5.5 (8.40)
*Prior medication, percent (number)* Anti-depressant Anti-epileptic Analgesic	92.9% (26) 46.4% (13) 32.1% (9)

*SD* standard deviation

### Effect of vitamin B12 on outcome measures

Total FIQR score and its domains including function, overall and symptoms improved significantly following the treatment (*p* value < 0.05). In addition, vitamin B12 could alleviate anxiety significantly; however, patients didn’t show any improvement in depression symptoms as well as pain VAS. Although the SF12 scores improved after treatment, these changes were not statistically significantly (*p* value > 0.05) (Table 2).

**Table 2:** Comparison of outcomes measures before and after treatment with vitamin B12

Variable	Before treatment	After treatment	Mean diff (95%C.I.)	*p* value
FIQR-total score	49.80 (21.86)	40.00 (18.36)	− 9.79 (− 4.13, − 15.45)	< 0.01
FIQR-function domain	13.17 (7.33)	10.30 (5.84)	− 2.86 (− 0.53, − 5.20)	0.01
FIQR-overall domain	10.32 (6.22)	8.25 (6.22)	− 2.07 (− 0.20, − 3.93)	0.03
FIQR-symptom domain	26.30 (10.39)	21.44 (8.58)	− 4.85 (− 1.91, − 7.79)	< 0.01
HADS-anxiety	9.33 (4.30)	7.70 (3.60)	− 1.63 (− 0.35, − 2.90)	0.01
HADS-depression	6.67 (3.90)	7.17 (3.76)	0.50 (1.60, − 0.60)	0.36
SF12-physical component	33.30 (10.22)	36.90 (12.30)	− 3.28 (9.56)	0.08
SF12-mental component	42.58 (14.70)	43.82 (14.39)	− 1.24 (10.15)	0.94
Pain-VAS	6.01 (2.68)	5.45 (2.71)	− 0.58 (− 1.51, 0.35)	0.21

Values are mean (standard deviation), *FIQR* revised fibromyalgia impact questionnaire, *HADS* hospital anxiety and depression scale, *SF*12 12-item short form survey, *VAS* visual analog scale

*p* value < 0.05 is statistically significant

### Model of change

The results of the GEE analysis are shown in Table 3. The result of the first model showed that changes in anxiety were significantly associated with changes in total FIQR scores (shown by time). The positive regression coefficient indicated that improvement in anxiety scores had a direct relationship with improvement in total FIQR improvement (B coefficient: 2.38 (CI:1.38–3.86); *p* value < 0.01). This model also revealed that even after adjusting for changes in anxiety, vitamin B12 reduced the FIQR scores (*p* value:0.03).

**Table 3:** Generalized estimating equation model for the treatment effect

Parameter	B	Standard error	95% confidence interval		*p* value
			Lower	Upper	
Model 1					
Time: before	6.36	3.03	0.41	12.31	0.03
Time: after*	Reference	–	–	–	–
Change in HADS_ Anxiety	2.38	0.51	1.38	3.38	< 0.01
Model 2					
Time: before	9.20	2.79	3.72	14.69	< 0.01
Time: after*	Reference	–	–	–	–
HADS-anxiety	0.93	0.63	− 0.30	2.17	0.14
HADS-depression	2.19	0.67	0.87	3.51	< 0.01
Marital status: married	12.10	4.53	3.22	20.98	< 0.01
Marital status: single	Reference	–	–	–	–
Age	− 0.21	0.28	− 0.78	0.34	0.45
Time from diagnosis	1.67	0.70	0.29	3.05	0.01

Model 1 is adjusted for change of anxiety and model 2 is adjusted for baseline anxiety, depression, marital status, age, and time from diagnosis

*HADS* hospital anxiety and depression scale

*This parameter is redundant

The second model showed that the improvement in total FIQR scores was not cofounded by patients’ baseline level of anxiety, depression, marital status, time from diagnosis, and age (*p* value: 0.01). The direction of regression coefficients indicated that FIQR scores improvement was associated with lower baseline depression, single status, and shorter duration of diagnosis (*p* value < 0.05) (Table 3).

## Discussion

This study showed that a short course of sublingual vitamin B12, 1000 mcg daily, can significantly improve the severity of FM as well as the anxiety score of FM patients. However, we did not find any improvement in the depression score and health status scales after vitamin B12 treatment.

It seems that vitamin B12 can potentially exert its analgesic effect and psychological modulation through several complex pathways. Preclinical studies have shown that cobalamin inhibits glutamate exocytosis, as an excitatory pain neurotransmitter, and when infused intracerebroventricularly, increases GABA cell contents, as an inhibitory pain neurotransmitter [6, 9]. The inhibitory effects of chronic exposure of vitamin B12 on glutamate-induced neurotoxicity were determined in cell culture, probably by altering the membrane properties through S-adenosylmethionine (SAM)-mediated methylation [26, 27]. SAM is one of the major methyl donors for methylation reactions throughout the body, including the methylation of myelin basic protein. Vitamin B12 in the form of methylcobalamin is required for the formation of methionine, known as a precursor of SAM, from homocysteine [28]. Furthermore, SAM is a precursor in the synthesis of melatonin and is also an enzymatic cofactor of glutathione production [29]. Melatonin has an antioxidant role, like glutathione. Studies show that SAM synthesis can enhance cognitive performance. SAM is an essential cofactor in the pathway of synthesis of epinephrine, which is involved in learning processes and memory consolidation [29, 30].

Moreover, increased homocysteine levels due to vitamin B12 deficiency could induce neurotoxicity caused by oxidative stress. Interestingly, Regland et al. showed the increased CSF level of homocysteine in FM patients and its correlation with patients’ fatiguability [16]. Increased level of homocysteine is also an independent risk factor for developing pain by inducing peripheral neuropathy [31]. Vitamin B12 also upregulates brain-derived neurotrophic factor (BDNF) and increases nerve conduction velocity, which may reflect part of the regeneration process and brain plasticity [32]. Another potential mechanism of action for the pain-reducing properties of vitamin B12 comes from interactions with prostaglandin synthesis, including cyclooxygenase (COX) enzymes. Animal studies showed simultaneous anti-inflammatory along analgesic effects on both peripherally and centrally induced pain models [10].

So, the preclinical and clinical studies showed that vitamin B12 is an essential micronutrient involved in the preservation of pain inhibitory and excitatory neurotransmitters balance, inflammation moderation, and consequently in the diverse behavioral process including sleep, learning, memory, and sensation of pain [7, 19]. Based on this, vitamin B12 could be an adjunctive therapy in FM patients who suffer from nociplastic pain and other central symptoms such as fatigue, sleep, and cognitive disturbance. To the best of our knowledge, no research has investigated the therapeutic effect of vitamin B12 on the disease impact and psychological profile of FM patients. Given the high proclivity of vitamin B12 to neural tissue and the fundamental role of this micronutrient in neuroplasticity, it would be conceivable to have a moderator effect on pain neurobiology and integrated psychological process. This is the first prospective study addressing this issue and supporting this viewpoint. Although mean pain VAS didn’t improve significantly in our FM patients after taking vitamin B12, the total and all domains of FIQR score improved significantly up to nearly 10 points. FIQR is a holistic indicator of FM impact in terms of pain, function, and other common symptoms of FM. The decrease in FIQR score to 10 points after vitamin B12 treatment in our study indicates significant improvement of the FM impact both statistically and clinically. Interestingly, the FIQR improvement was independent of demographic and psychological profiles and preserved after adjusting for these potential confounding factors. Congruent with our result, Regland et al. showed positive dose–response and long-lasting effects of vitamin B12 injection in FM/chronic fatigue syndrome (CFS). They compared the patients (good responders versus mild responders) who had already been taking vitamin B12 shots for at least six months to twenty years [17]. In this study of FM/chronic fatigue syndrome, patients who were good responders were found to be using a higher and more frequent doses of vitamin B12 for a longer period [17]. Decrease in pain scores after vitamin B12 supplementation have been also reported in other types of pain such as peripheral neuropathy and post-chemotherapy musculoskeletal pain. Although pain mechanisms are different in neuropathic or nociceptive pain, vitamin B12 as a pain killer has had positive effects in these studies, similar to our studied FM patients [11, 12].

Interestingly, despite promising reports of vitamin B12 effect on various types of pain, existing studies has implied that serum levels of vitamin B12 in FM patients are not altered in comparison with control group [13]. In Regland et al.’s study, all patients had normal serum levels of vitamin B12 and homocysteine, however, levels of B12 in CSF correlated significantly with fatigue and items of a psychopathological scale [16]. This discrepancy indicates a correlation between central vitamin B12 levels, not peripheral/serum level, with pain and fatigue. There is scarce data on this issue, thereby vitamin B12 measurement is not recommended for FM patients.

Although our results were promising in reducing anxiety scores after vitamin B12 treatment, the patients’ depression scores were not diminished post-treatment in our patients. However, this finding doesn’t indicate the ineffectiveness of vitamin B12 in the treatment of depression. It may be because of the short treatment course in our study (50 ± 2 days) or continuing of FM medications such as duloxetine or tricyclic antidepressants in the study time which could attenuate the vitamin B12 effect. Previous data support the role of vitamin B12 supplementation in preventing or improving depressive symptoms and cognitive dysfunction [19]. Given this, we surmise the achievement of positive effects need the application of vitamin B12 in a larger population and with a longer treatment duration.

Interestingly, lower baseline depression, single status, and shorter duration of diagnosis are significantly correlated with FIQR scores improvement after vitamin B12 treatment. These baseline characteristics favor the effectiveness of vitamin B12 treatment. It may indicate some psychological status, and demographic characteristics may influence the treatment response.

Our study had some limitations. First, only female patients from one tertiary care center with a relatively small sample size were recruited; thus, the study findings cannot be extrapolated to the general FM population. Secondly, we did not have a placebo arm to compare with the treatment arm. Providing the placebo form of sublingual vitamin B12 similar to the original drug was very difficult. So, we decided the study design be based on the pre-post intervention model. Although the study design is not randomized, the results of this type of study as a quasi-experimental study could still give us a relatively high level of evidence. Thirdly, this study was an open label trial delimiting our results for the interpretation with high confidence. Furthermore, we used vitamin B12 as an adjuvant drug with patients’ current treatment regimen such as FDA-approved FM drugs. It may change or attenuate the effect of vitamin B12 on the outcomes. So, future randomized control trial needs to address this potential confounding factor in evaluating more realized vitamin B12 effect. Finally, our study duration was short and approximately two months. Given the safety of vitamin B12, it would be more favorable to treat FM patients with a longer duration to achieve a larger effect on pain and psychological illness improvement. It appears that long-term treatment for the moderation of the nociplastic alterations in the FM nervous system is needed.

This study can be a node for developing more mature and controlled studies to evaluate the effect of vitamin B12 as a safe and potentially important moderator of nociplastic pain and psychological illness in FM. This prospective study was the first study trying to clarify the effect of vitamin B12 in FM patients regarding symptom severity and psychological profiles.

## Conclusion

Considering the promising effects of vitamin B12 in improving disease severity and anxiety of FM patients as well as the absence of any side effects in the studied dosage, we postulate that vitamin B12 has a strong potential to consider, at least, as an adjunctive therapy of FM.

## Data Availability

The datasets generated and/or analyzed during the current study are not publicly available due to confidentiality issues but are available from the corresponding author on reasonable request.
